# CMRA-DETR: a lightweight and high-accuracy detection framework for MRI-based brain tumor identification

**DOI:** 10.3389/fmed.2026.1839485

**Published:** 2026-05-04

**Authors:** Cai Weng, Bowei Huang, Jinghui Chen, Wei Hu, Zhiqing Huang, Punan Weng, Hongjia Zhao, Minqin Zheng

**Affiliations:** 1 The Second Affiliated Hospital of Fujian University of Traditional Chinese Medicine, Fuzhou, Fujian, China; 2 Fujian University of Traditional Chinese Medicine, Fuzhou, China; 3 The First Clinical Medical College, The Affiliated People’s Hospital of Fujian University of Traditional Chinese Medicine, Fuzhou, Fujian, China; 4 The Third Affiliated People’s Hospital of Fujian University of Traditional Chinese Medicine, Fuzhou, Fujian, China; 5 Fuzhou Hospital of Traditional Chinese Medicine, Fuzhou, Fujian, China

**Keywords:** artificial intelligence, brain tumor, magnetic resonance imaging, object detection, RT-DETR

## Abstract

**Introduction:**

Brain tumor lesions in MRI images are characterized by low contrast, indistinct boundaries, and irregular morphology, posing substantial challenges for automated detection. RT-DETR provides an end-to-end, NMS-free detection paradigm, but its original design is optimized for natural images and exhibits three domain-specific limitations in brain tumor MRI detection: insufficient local texture perception of low-contrast lesion boundaries, neglect of feature magnitude differences in linear attention, and weak modeling of spatial continuity for morphologically irregular tumors. This study proposes CMRA-DETR (CSP-MambaOut with Retention and Magnitude-Aware Attention for Real-Time Detection Transformer), a lightweight yet high-accuracy detection framework built upon RT-DETR-R18, to address these limitations through targeted architectural adaptations.

**Methods:**

CMRA-DETR introduces three improvements: (1) a CSP-MambaOut backbone that enhances local texture perception of low-contrast lesion boundaries via gated feature selection; (2) an AIFI-MALA module that introduces magnitude-aware linear attention to correct the distributional smoothing deficiency of standard linear attention; and (3) a RetBlockC3 module that incorporates a Manhattan distance decay-based spatial retention mechanism to improve modeling of spatial continuity for morphologically irregular tumors. The model was trained and evaluated on an internal independent test set of 5,731 MRI images covering four categories (no tumor, meningioma, glioma, and pituitary adenoma), and further assessed on an external independent test set from the BRISC dataset without fine-tuning. Its performance was benchmarked against Faster R-CNN, YOLOv5n, YOLOv8n, YOLO11n, YOLO12n, and the RT-DETR-R18 baseline.

**Results and discussion:**

On the internal independent test set, CMRA-DETR achieved achieves *p* = 95.5%, *R* = 95.7%, mAP@50 = 97.9%, and mAP@50-95 = 82.6%, while reducing parameters and GFLOPs by 37.7% and 30.9%, respectively, relative to the baseline, achieving competitive or superior performance compared to the evaluated baseline models under the specified experimental conditions. On the external independent test set from the BRISC dataset, the model achieved mAP@50 = 96.6% and mAP@50-95 = 79.8% without fine-tuning, demonstrating robust cross-dataset generalization. These findings indicate that CMRA-DETR achieves a favorable balance among detection accuracy, model lightweightness, and inference efficiency, demonstrating practical potential for AI-assisted brain tumor detection on resource-constrained clinical devices.

## 1 Introduction

Brain tumors are neoplasms formed by the aberrant proliferation of cells within the intracranial tissue. According to the World Health Organization (WHO) classification system, brain tumors are graded from Grade I to Grade IV, with malignancy increasing with grade (1). The most common primary brain tumors encountered clinically include meningiomas, gliomas, and pituitary adenomas. Among these, gliomas carry the highest degree of malignancy and account for approximately one-third of all primary brain tumors (2, 3). Consequently, achieving early and accurate detection and classification of brain tumors is of paramount clinical importance for guiding therapeutic decision-making and improving patient outcomes.

Magnetic resonance imaging (MRI) has become the primary modality for clinical brain tumor diagnosis, owing to its superior soft-tissue contrast, high spatial resolution, and freedom from ionizing radiation (4, 5). MRI enables comprehensive assessment of tumor characteristics through multi-planar and multi-sequence imaging (6, 7). Nonetheless, automated analysis of brain MRI images faces considerable challenges, including signal heterogeneity, irregular tumor morphology, indistinct boundaries, and acquisition artifacts, underscoring the clinical value of developing efficient and reliable automated detection algorithms.

Traditional detection approaches based on handcrafted features are ill-suited to the complex patterns in brain tumor MRI images (8, 9). In recent years, deep learning methods—particularly convolutional neural networks (CNNs)—have achieved substantial advances in brain tumor detection and classification. For classification tasks, Swati et al. (10) and Deepak and Ameer (11) demonstrated the effectiveness of transfer learning and pretrained CNN features for brain tumor MRI classification. Rehman et al. (12) compared several mainstream CNN architectures on brain tumor MRI classification benchmarks. In object detection, Sajjad et al. (13) validated two-stage detectors for brain tumor localization. Abiwinanda et al. (14) explored lightweight CNN architectures for brain tumor MRI classification for reducing overfitting under limited annotations. Nevertheless, mainstream CNN-based detectors such as Faster R-CNN retain inherent limitations, including dependence on large annotated datasets, insufficient sensitivity to small lesions, anchor hyperparameter sensitivity, and reliance on non-maximum suppression (NMS) that complicates end-to-end deployment.

To overcome the fixed receptive fields of CNNs and their limited ability to capture long-range dependencies, Transformer-based methods have been progressively introduced into brain tumor MRI detection and classification. Khaliki and Başarslan (15) adapted pretrained Vision Transformer (ViT) models via transfer learning for brain tumor MRI classification. Martín and Sánchez (16) demonstrated hierarchical window attention for brain tumor MRI with cross-domain generalizability on multi-center MRI data. Asiri et al. (17) examined Transformer transfer learning under few-shot brain tumor MRI classification scenarios. Despite these advances, the quadratic time complexity of global self-attention remains a significant computational bottleneck for real-time deployment.

RT-DETR (Real-Time Detection Transformer) has emerged as a promising end-to-end detection framework that eliminates NMS post-processing through an anchor-free mechanism. Its Efficient Hybrid Encoder decouples multi-scale feature processing into intra-scale interaction and cross-scale fusion, integrating the complementary strengths of CNN local feature extraction and Transformer global context modeling while maintaining real-time inference speed. However, RT-DETR’s original design is optimized for natural image scenarios and has not been specifically adapted to the distinctive characteristics of medical imaging.

When RT-DETR is applied to brain tumor MRI detection, its original architecture reveals several domain-specific inadequacies. First, in the feature extraction stage, the ResNet backbone is optimized for natural images, exhibiting limited sensitivity to local textures in low-contrast lesion regions and blurred tumor boundaries in MRI scans, resulting in insufficient feature representation. Second, in the encoder’s intra-scale interaction module (AIFI), standard self-attention assigns similar weights to features at different spatial positions regardless of their magnitude, whereas brain tumor regions are often identified based on specific signal amplitude distributions; neglecting magnitude differences weakens the model’s ability to discriminate lesion areas. Third, the encoder’s cross-scale feature aggregation module (RepC3) lacks explicit modeling of the spatial continuity and local positional relationships of tumor lesions, making it difficult to fully capture the spatial structural information of morphologically irregular tumors. These three deficiencies collectively constrain the performance of RT-DETR in brain tumor MRI detection.

To address these limitations, this study proposes CMRA-DETR (CSP-MambaOut with Retention and Magnitude-Aware Attention for Real-Time Detection Transformer), built upon the lightweight RT-DETR with a ResNet18 backbone. Rather than introducing entirely new theoretical frameworks, the principal contributions lie in the targeted adaptation and integration of existing mechanisms to the specific challenges of brain tumor MRI detection, as follows:

1. A CSP-MambaOut lightweight backbone is constructed by integrating MambaOut gated convolutional blocks with a Cross Stage Partial Network (CSP) structure. This design strengthens the backbone’s local texture perception of low-contrast lesion regions and blurred boundaries in a computationally efficient manner.
2. A Magnitude-Aware Linear Attention (MALA) mechanism is introduced into the encoder’s intra-scale interaction module AIFI, constructing an AIFI-MALA module. By explicitly modeling feature magnitude information, MALA corrects the distributional smoothing deficiency inherent in standard linear attention, thereby enhancing the model’s discriminative sensitivity to lesion signal characteristics.
3. A RetBlockC3 spatial retention feature aggregation module is constructed by incorporating RetBlock (Retention Block) into the encoder’s cross-scale aggregation module RepC3. The Manhattan distance decay-based spatial retention mechanism introduces an explicit spatial positional prior into the feature aggregation process, enhancing the modeling of spatial continuity for morphologically irregular tumors.

## 2 RT-DETR

RT-DETR (Real-Time Detection Transformer) is an end-to-end object detection framework proposed by Zhao et al. (18). Its core concept follows DETR’s set prediction paradigm, employing an anchor-free detection mechanism that eliminates the NMS post-processing step and directly outputs final detection results. To achieve a balance between real-time performance and detection accuracy, RT-DETR introduces an Efficient Hybrid Encoder that decouples multi-scale feature processing into two stages—intra-scale interaction and cross-scale fusion—thereby substantially improving multi-scale information modeling efficiency. Architecturally, RT-DETR consists of four components: a Backbone, a Hybrid Encoder, a Transformer Decoder, and a detection prediction head. The Backbone extracts multi-level semantic features from the input image, producing multi-scale outputs from each stage. The Hybrid Encoder performs intra-scale information interaction and cross-scale fusion on these multi-scale features to enhance global contextual representation. The Decoder employs a set of learnable object queries, progressively aggregating object-relevant information via attention mechanisms, with the prediction head simultaneously outputting class probabilities and bounding box regression results. Additionally, RT-DETR employs an IoU-aware query selection strategy to improve the matching quality between queries and ground-truth targets.

Brain tumors in MRI images commonly present as morphologically irregular lesions with complex boundary transitions and pronounced intra-tumoral signal heterogeneity, and their localization and classification must be performed simultaneously within a unified framework (19, 20). RT-DETR’s global context modeling capability facilitates more complete characterization of lesion spatial relationships against a complex tissue background, while its end-to-end, NMS-free output mechanism enables a structurally clean detection pipeline that is amenable to clinical integration and supports flexible speed-accuracy tradeoffs across different hardware platforms by adjusting the number of decoder layers. These properties make RT-DETR a more suitable foundation for brain tumor MRI detection than traditional anchor-based detectors, and constitute the primary rationale for its selection as the base framework in this study.

**Figure 1:**
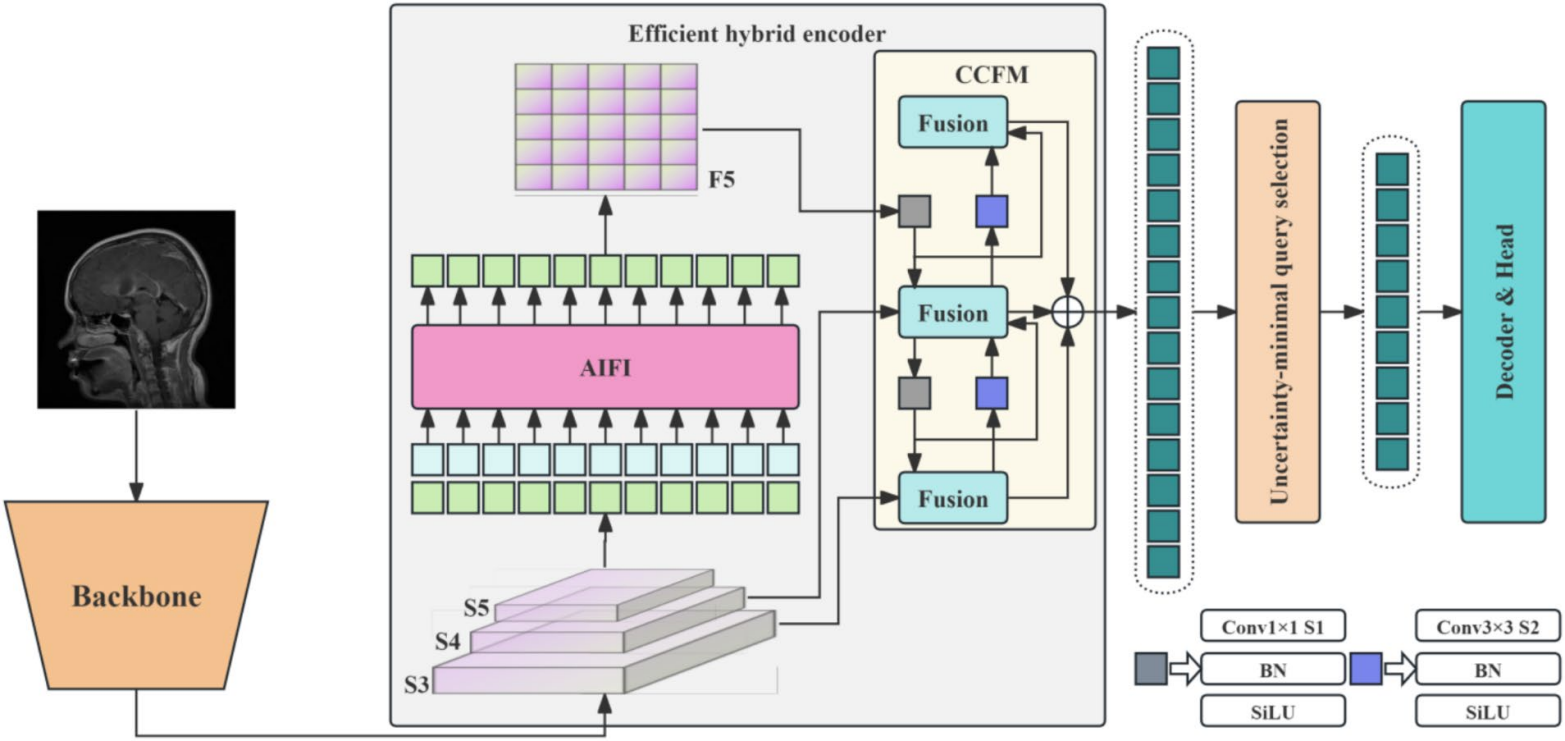
Architectural diagram of the RT-DETR model.

RT-DETR is available in multiple variants employing ResNet18, ResNet34, ResNet50, and ResNet101 backbones. To accommodate resource-constrained hardware, this study adopts the ResNet18-based model, which has the smallest parameter count and memory footprint, as the baseline for subsequent improvements. The baseline model architecture is illustrated in Figure 1.

## 3 CMRA-DETR

**Figure 2:**
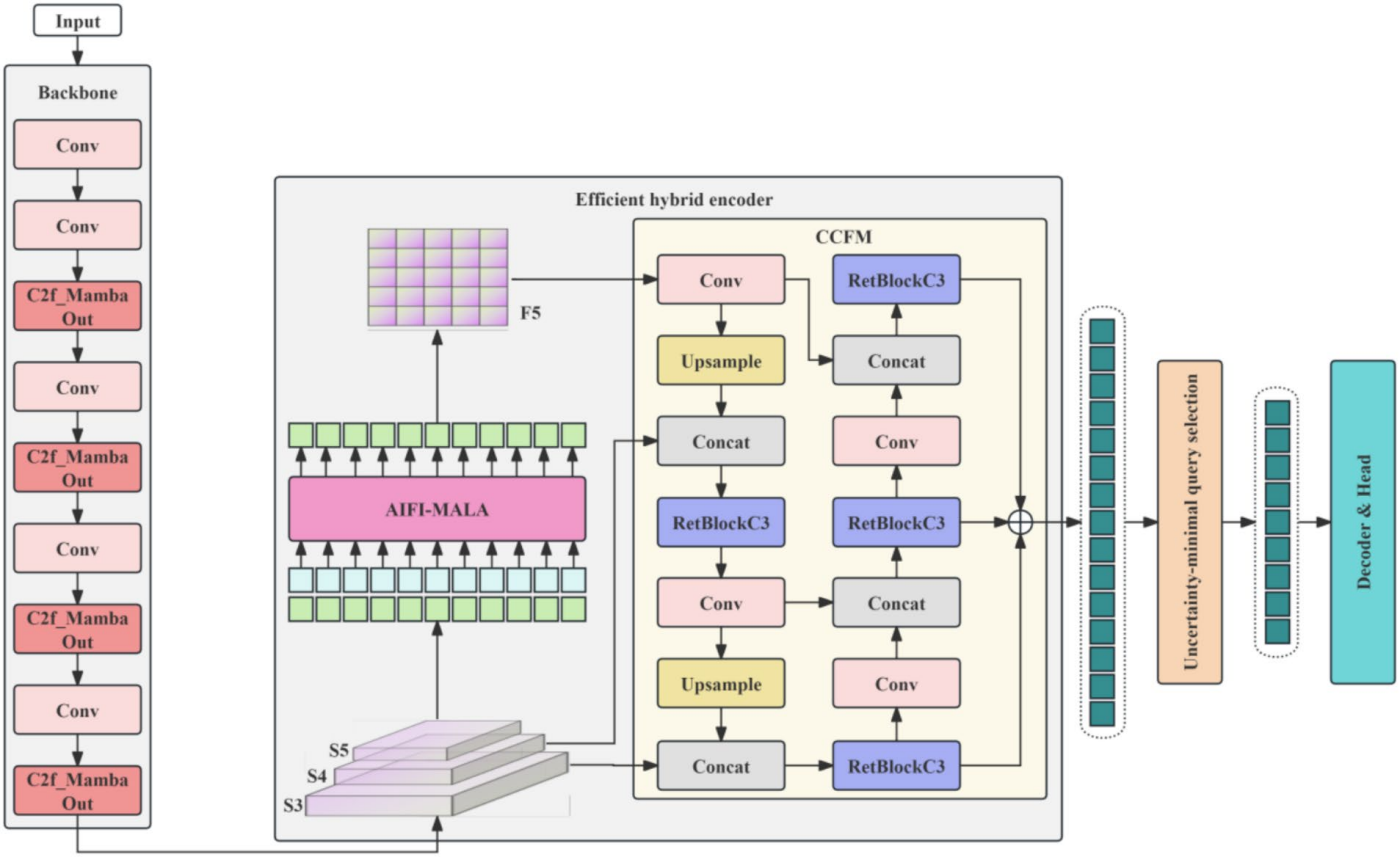
Architectural diagram of the CMRA-DETR model.

The proposed CMRA-DETR is built upon the RT-DETR-R18 framework and introduces targeted improvements at three key locations—the backbone, the encoder intra-scale interaction module (AIFI), and the encoder cross-scale aggregation module (RepC3)—to address the three principal limitations of RT-DETR in brain tumor MRI detection. The overall network architecture is illustrated in Figure 2. The following three subsections describe the design rationale and implementation details of each improvement module.

### 3.1 CSP-MambaOut lightweight backbone

The baseline model employs a ResNet18 backbone, which offers compact parameter size and high inference efficiency. However, repeated convolution and pooling downsampling operations progressively erode edge detail information during feature extraction, causing local texture features to attenuate across hierarchical layers. This deficiency is particularly pronounced in brain tumor MRI detection: tumor regions are generally characterized by low contrast and indistinct boundaries, and the loss of shallow-level detail directly diminishes the model’s sensitivity to lesion boundaries and fine-grained textural structures. Moreover, although ResNet18’s residual connections alleviate the vanishing gradient problem to some extent, its fixed channel information flow path lacks inter-stage feature bifurcation and fusion mechanisms, limiting its capacity to model the heterogeneous internal structures of tumors and constraining local feature representation for morphologically irregular lesions.

**Figure 3:**
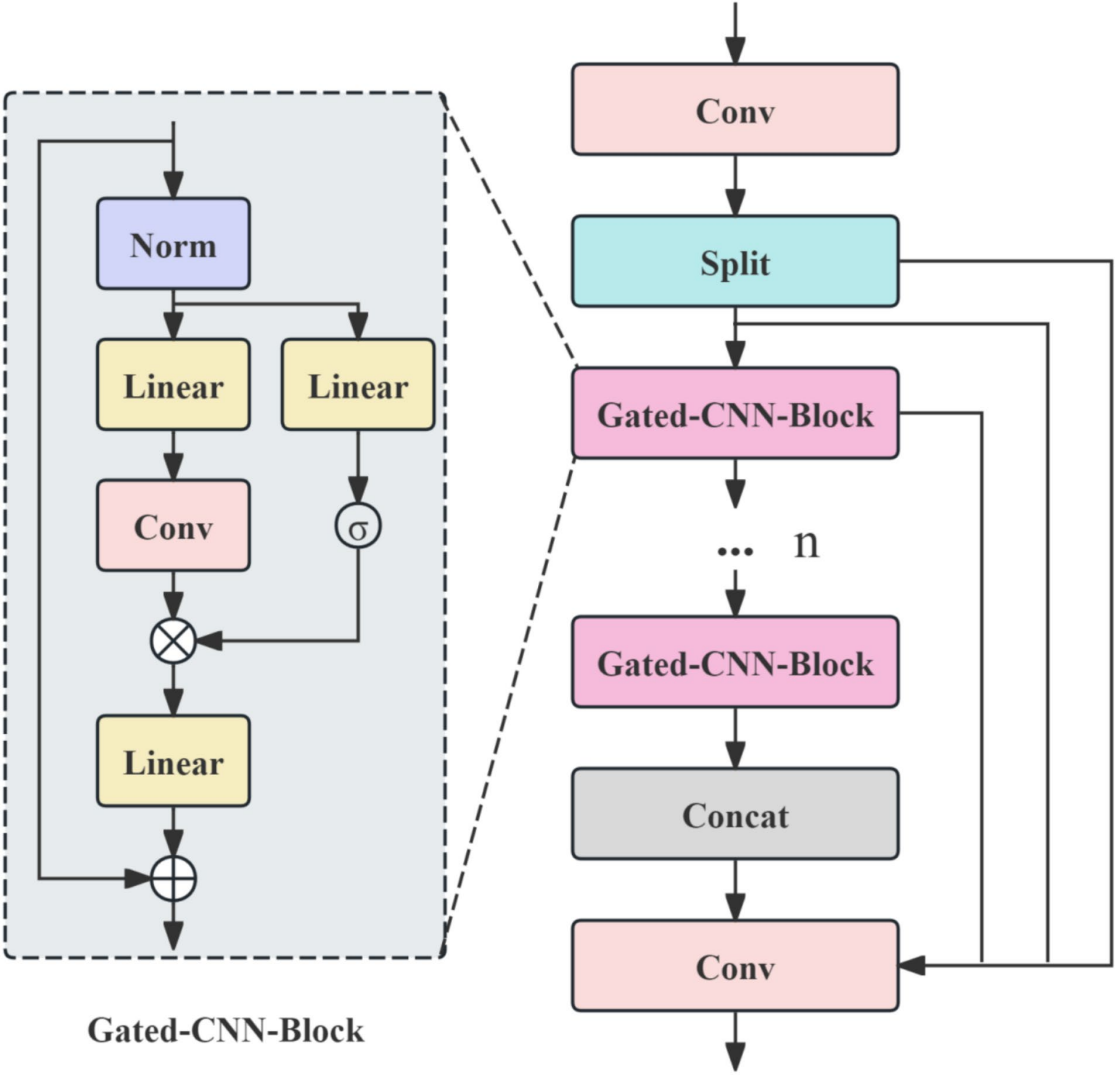
CSP-MambaOut network architecture.

To address these limitations, this study proposes CSP-MambaOut, a lightweight backbone network that combines the Cross Stage Partial (CSP) structure (21) with MambaOut modules (22). The network architecture is depicted in Figure 3. The CSP structure employs staged feature flow and fusion mechanisms to effectively reduce feature redundancy and enhance gradient flow while preserving shallow-level spatial detail information under lightweight deployment conditions, facilitating the capture of low-contrast textural cues in boundary regions of brain tumors. The MambaOut gated dynamic feature module performs dynamic feature selection through a gating mechanism, improving the network’s perceptual sensitivity to complex intra-tumoral texture variations and indistinct boundary regions without substantially increasing computational cost.

Specifically, the input features are first encoded through a convolutional layer. The features are then bifurcated along the channel dimension in a Split module into a residual direct-connection branch and a main processing branch. The residual branch preserves the original spatial information, while the main branch is composed of multiple stacked Gated-CNN-Blocks, each integrating normalization, linear transformation, convolution, and gating mechanisms to achieve dynamic feature selection and information enhancement. After progressive processing through multiple Gated-CNN-Blocks, the high-level semantic features extracted by the main branch are concatenated with the shallow spatial information from the residual path along the channel dimension in a Concat module, and the merged features are integrated and output by a final convolutional layer. The gated feature selection process is formalized in Equation (1):


$$y=x+\sigma (W_{g}\times \text{Norm}(x))\circ \text{conv}(W_{f}\times \text{Norm}(x))$$ (1)

Where x denotes the input feature, σ is the Sigmoid activation function, ∘ denotes the Hadamard product, and $W_{g}$ and $W_{f}$ are the linear transformation weight matrices of the gating branch and the feature branch, respectively.

In summary, CSP-MambaOut employs channel bifurcation, gated feature selection, and residual fusion within a lightweight framework to effectively preserve shallow spatial detail and enhance local texture perception of low-contrast lesion regions, thereby establishing a strong representational foundation for precise encoding.

### 3.2 AIFI-MALA magnitude-aware feature interaction module

The AIFI structure in RT-DETR—a lightweight linear attention and convolution hybrid unit for intra-scale multi-feature interaction—is prone to feature distribution smoothing and insufficient response contrast when processing brain tumor MRI images. This deficiency is especially prominent in medical imaging: brain tumor regions are typically identified based on specific signal amplitude distributions, yet standard linear attention, after kernel function mapping, discards the magnitude information of query vectors. This causes the resulting attention distribution to be excessively smooth, making it difficult to effectively distinguish signal differences between lesion and normal brain tissue, performing markedly worse than Softmax attention and ultimately impairing the model’s discriminative ability for low-contrast lesions and blurred boundary regions.

**Figure 4:**
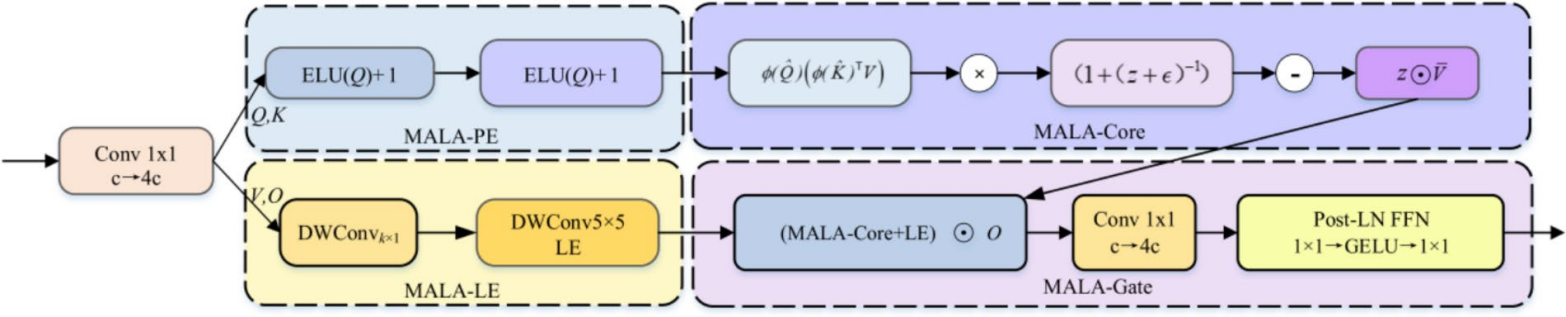
Architecture of the AIFI-MALA module.

To this end, this study introduces the AIFI-MALA module, whose network architecture is shown in Figure 4. It comprises four complementary submodules: MALA-Core (magnitude-corrected kernel attention), MALA-PE (dual-axis rotary phase relative positional encoding), MALA-LE (depthwise separable local enhancement position component), and MALA-Gate (pointwise output gating). AIFI-MALA replaces the interaction core of AIFI with magnitude-aware linear attention without increasing parameter count or computational cost. It embeds relative positional information multiplicatively into the Q/K inner product space via axial RoPE positional encoding, achieves lightweight local enhancement, and finally fuses the attention output with a gating component via pointwise multiplication—enabling the model to simultaneously focus on lesion details and maintain global signal structure in MRI images, effectively alleviating the attention uniformity problem and enhancing discriminative sensitivity to tumor lesion signal characteristics.

Given the input feature $X\in R^{B\times C\times H\times W}$ , Q,K,V , and O are generated simultaneously via a single 1 × 1 convolution, and the positive-valued kernel mapping ϕ(·)=ELU(·)+1 is adopted, as defined in Equation (2):


$$\begin{array}{l} Q,K,V,O={\text{Conv}}_{1\times 1}(X),\phi (Q)=\mathrm{ELU}(Q)+1,\\ \;\;\;\;\;\;\;\;\;\;\;\;\;\;\;\;\;\;\;\;\;\;\;\;\;\;\;\;\;\;\;\phi (K)=\mathrm{ELU}(K)+1 \end{array}$$ (2)

In MALA-PE, dual-axis rotary RoPE is applied to Q and K for each attention head. Frequency vectors $\omega_{h}$ and $\omega_{w}$ are constructed for the height and width axes, respectively, and axial (pairwise-channel) complex-plane rotation transforms are applied to yield $\hat{Q}$ and $\hat{K}$ , embedding relative positional information into the inner product space without additional operators, as expressed in Equation (3):


$$\begin{array}{c} \begin{array}{l} {\text{RoPE}}_{2D}(t;h;w)=t⊙\cos(\theta_{h,w})+\text{shift}(t)⊙\sin(\theta_{h,w}),\\ \;\;\;\;\;\;\;\;\;\;\;\;\;\;\;\;\;\;\;\;\;\;\;\;\;\;\;\;\;\;\;\;\;\;\;\;\;\;\;\;\;\;\;\theta_{h,w}=[h\omega_{h};w\omega_{w}] \end{array} \end{array}$$ (3)

Where ${\text{RoPE}}_{2D}$ ( · ) is the 2D rotary positional encoding map, t is the positional vector to be rotated, $\theta_{h,w}$ is the positional angle vector, and shift(t) is an intra-vector channel-shift operation. This 2D axial RoPE accounts for anisotropic decay along both row and column directions, making it particularly suited to the asymmetric signal distribution of tumor regions along different spatial axes in brain MRI images. To explicitly address the magnitude neglect problem, a correction factor and bias computation that are explicitly sensitive to the query magnitude are introduced to form the magnitude self-consistent kernel attention of MALA-Core, as formulated in Equation (4). Let $\bar{K}=\frac{1}{\mathrm{HW}}\sum_{i}K_{i},\bar{V}=\frac{1}{\mathrm{HW}}\sum_{i}V_{i},z=\phi (\hat{Q}){\bar{K}}^{⊤}$ ; then:


$$\begin{array}{c} \begin{array}{l} \text{MALA}-\text{Core}(Q,K,V)=\phi (\hat{Q})[\phi {\left(\hat{K}\right)}^{⊤}V]\\ ⊙[1+{\left(z+\varepsilon \right)}^{-1}]-z⊙\bar{V} \end{array} \end{array}$$ (4)

Where $K_{i}$ is the key vector at position i ; $\bar{K}$ and, $\bar{V}$ are global representative vectors obtained by spatial average pooling; the first term is the kernelized product of standard linear attention; ϵ is a small constant for numerical stability $\epsilon =10^{-6}$ ; ${\left(z+\epsilon \right)}^{-1}$ re-weights the query magnitude to recover a Softmax-like sparse, balanced score distribution; and the second term subtracts a magnitude-proportional global mean bias from the output, theoretically correcting the distributional smoothing and contrast insufficiency caused by magnitude neglect in linear attention, thereby helping the model maintain stable lesion discriminability within the signal-heterogeneous internal structure of tumors.

For positional enhancement, MALA-LE employs a 5 × 5 depthwise separable convolution grouped by C to generate a locally learnable positional perturbation DW-LPE(V), capturing local textures and fine boundary features of tumor regions without cross-channel mixing. Finally, MALA-Gate introduces pointwise multiplicative gating to fuse the kernel attention output with the gating component O, projected back to C dimensions via a 1 × 1 convolution, as given in Equation (5):


$$\begin{array}{c} Y={\text{Proj}}_{1\times 1}\{[\text{MALA}-\text{Core}(Q,K,V)+\mathrm{DW}-\mathrm{LPE}(V)]⊙O\} \end{array}$$ (5)

Where $\;{\text{Proj}}_{1\times 1}$ ( · ) denotes 1 × 1 linear projection. A post-normalization two-layer feed-forward network with GELU activation then performs the residual update, as shown in Equation (6):


$$\begin{array}{c} Y^{\prime }=\mathrm{LN}(X+\mathrm{LN}\{Y+{\text{Conv}}_{1\times 1}[\text{GELU}({\text{Conv}}_{1\times 1}(Y))]\}) \end{array}$$ (6)

Through the synergistic design of magnitude-aware linear attention, dual-axis RoPE positional encoding, and gated output, AIFI-MALA effectively alleviates the attention uniformity problem of the original AIFI while maintaining real-time inference capability, significantly enhancing the model’s discriminative sensitivity to low-contrast lesion signal characteristics.

### 3.3 RetBlockC3 spatial retention feature aggregation module

**Figure 5:**
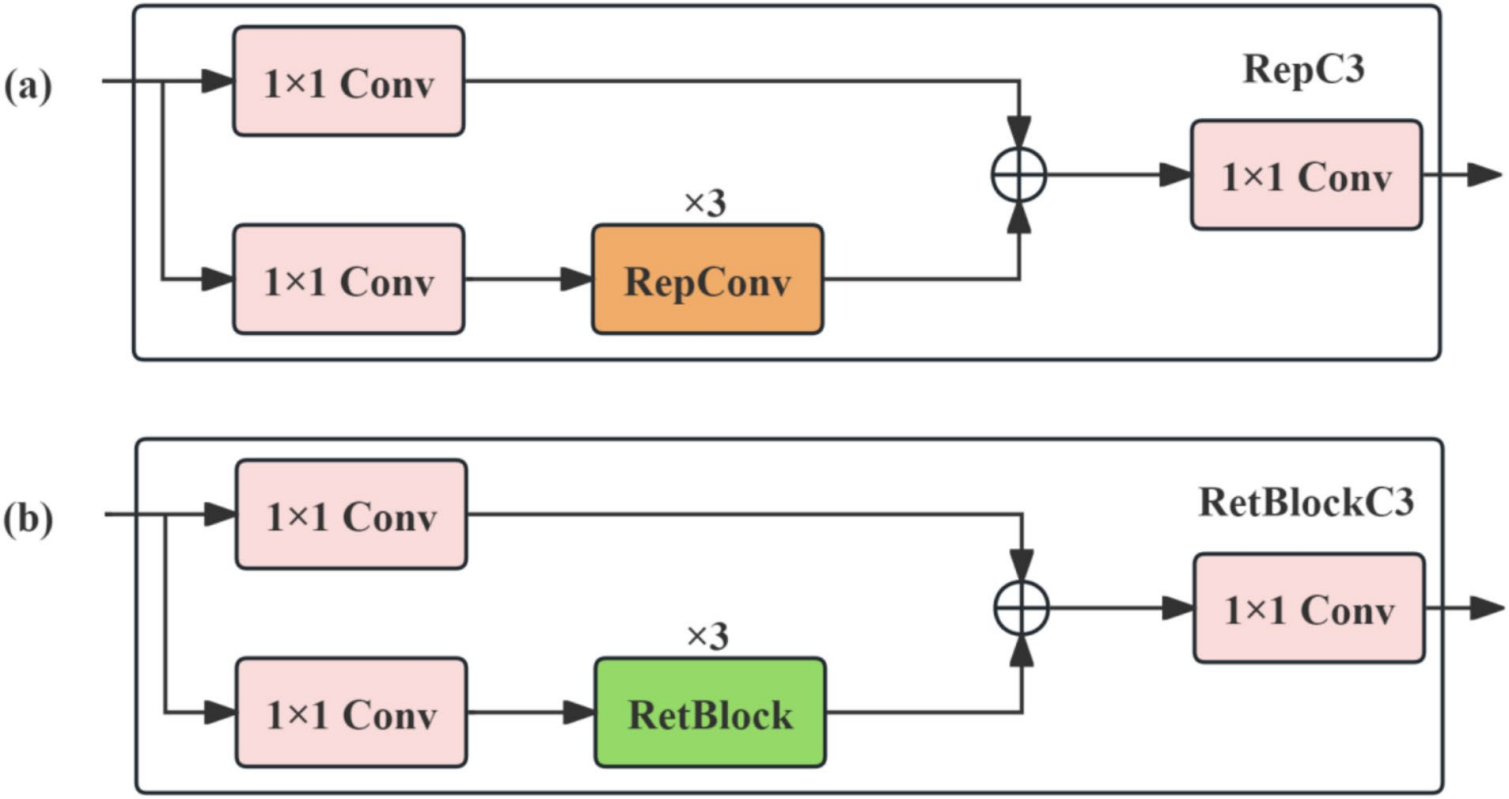
**(a)** Structural diagram of RepC3. **(b)** Structural diagram of RetBlockC3.

The cross-scale feature aggregation network in RT-DETR employs the RepC3 module for feature processing, as illustrated in Figure 5a. This module first applies batch normalization to the input feature map, then extracts spatial features via convolution. In the feature fusion stage, the convolution-extracted features are element-wise summed with the batch-normalized input, followed by a SiLU activation function for nonlinear transformation. However, RepC3 exhibits two fundamental limitations in brain tumor MRI detection. On one hand, its channel aggregation process relies solely on the local receptive field of convolution and lacks explicit modeling of the spatial continuity and local positional relationships of tumor lesions, making it difficult to establish long-range dependencies among spatial positions within lesion regions and insufficiently capturing the spatial structural information of morphologically irregular, boundary-complex tumors. On the other hand, in the face of complex pathological scenarios involving blurred tumor boundaries, highly heterogeneous morphology, and low contrast with surrounding soft tissue, its conventional convolutional feature extraction fails to meet the high demands of medical imaging for fine-grained lesion perception, particularly constraining accurate detection of aggressively infiltrative, morphologically heterogeneous lesions such as gliomas. Together, these limitations degrade the overall quality of multi-scale feature fusion.

To address these limitations, this study introduces RetBlock (23)—the core module from RMT (Retentive Networks Meet Vision Transformers)—and proposes the RetBlockC3 spatial retention feature aggregation module by replacing RepConv in RepC3 with RetBlock. This substitution introduces an explicit spatial positional prior into the feature aggregation process, enhancing the modeling of spatial continuity for morphologically irregular tumors while maintaining computational efficiency.

**Figure 6:**
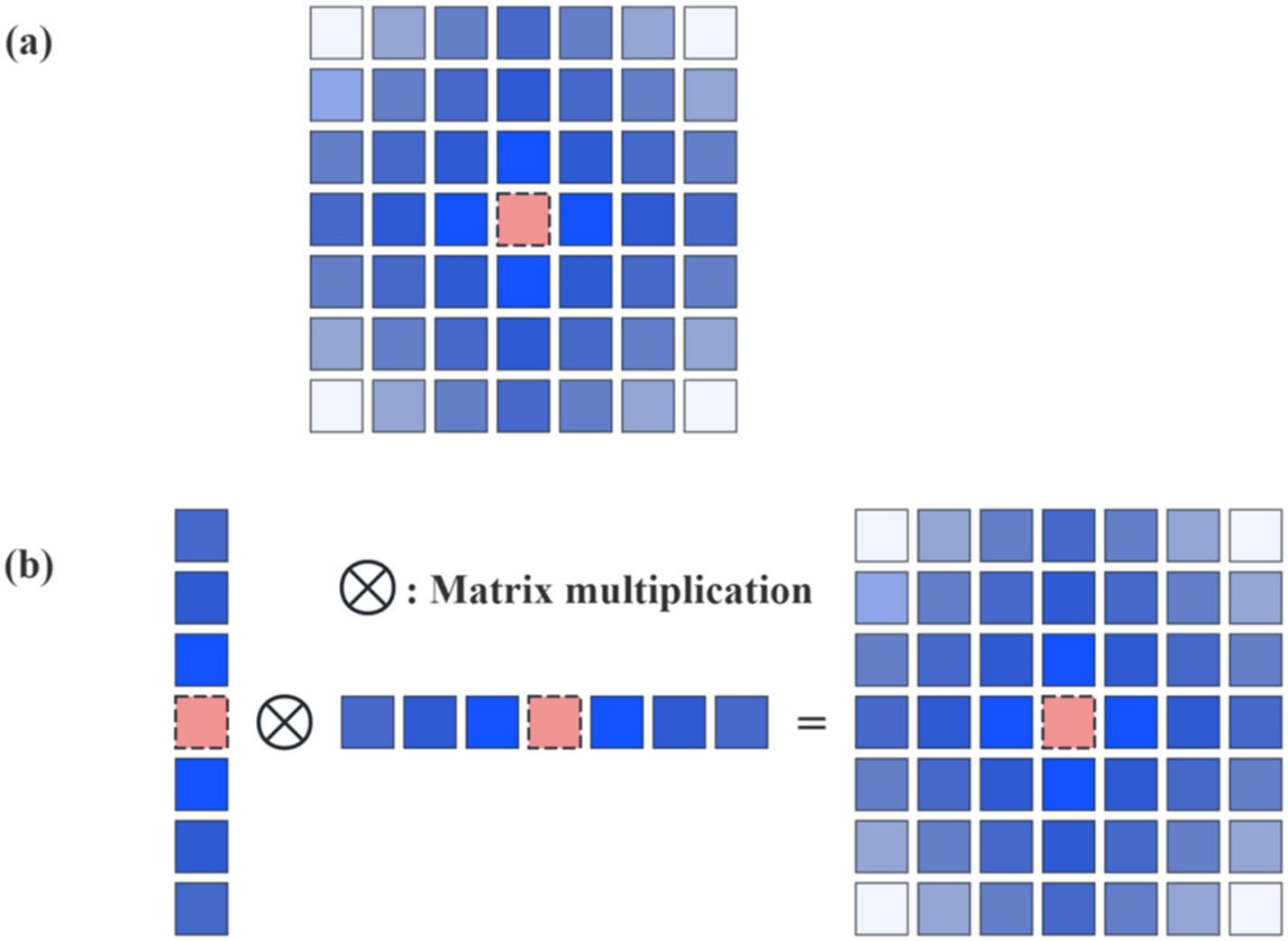
**(a)** Manhattan self-attention. **(b)** Decomposed Manhattan self-attention.

The core operating principle of RetBlock is as follows. It first integrates the retention mechanism of Retentive Networks with multi-head Manhattan Self-Attention (MaSA) and adjusts attention weights using a spatial decay matrix, as illustrated in Figure 6a. Second, a self-attention decomposition strategy is adopted (Figure 6b), decomposing 2D attention into 1D horizontal and vertical attention, each applying Manhattan distance-based decay matrices. This achieves global information modeling at linear complexity without disrupting the spatial decay structure, effectively reducing computational cost. Third, a Local Context Enhancement (LCE) module, implemented with depthwise separable convolutions, is introduced to strengthen the model’s spatial perception of local boundary textures in tumor lesions. Additionally, 2D relative positional encoding (RelPos2d) is introduced to provide precise spatial positional information to the attention mechanism, further improving spatial positional modeling accuracy.

The specific network architecture of RetBlockC3 is shown in Figure 5b. The fusion pipeline proceeds as follows: the higher-level semantic feature S5, after processing by the AIFI-MALA module, is combined with shallower features S3 and S4 as joint inputs to the CCFM module for multi-scale fusion. The CCFM module achieves multi-scale feature integration by embedding multiple RetBlockC3 fusion blocks along the fusion path—each fusion block employs a dual-path design containing two 1 × 1 convolutions for channel dimension adjustment, with RetBlock performing feature fusion, and the outputs of the two paths are aggregated by element-wise addition. The central role of RetBlockC3 lies in its Manhattan distance decay-based spatial retention mechanism, which introduces an explicit spatial positional prior into feature aggregation, enabling the model to establish spatial associations among positions within lesion regions during multi-scale feature fusion and thereby more completely characterizing the spatial continuity of morphologically irregular tumors. The LCE module further strengthens the capture of local boundary details of tumors, compensating for global attention’s limited sensitivity to local textures.

RetBlockC3, through the integration of a Manhattan distance decay-based spatial retention mechanism and local context enhancement, effectively compensates for the inherent deficiencies of RepC3 in spatial structure modeling and fine-grained lesion perception, providing the decoder with fused features of substantially stronger spatial structural representation for precise localization and classification.

## 4 Experiments and results

### 4.1 Data acquisition

The medical imaging data used in this study were sourced from two distinct datasets. Dataset 1 is a publicly available brain tumor MRI dataset released on the Kaggle platform (24, 25), comprising 5,731 MRI images across four categories: no tumor (*n* = 880), meningioma (*n* = 1,738), glioma (*n* = 1,408), and pituitary adenoma (*n* = 1,705); this dataset was used for model training, validation, and internal testing to evaluate model performance. Dataset 2 is derived from the publicly available BRISC dataset (26), comprising 512 MRI images across the same four categories (128 images per category: no tumor, meningioma, glioma, and pituitary adenoma), and was used as an external independent test set for evaluating model generalization performance.

**Figure 7:**
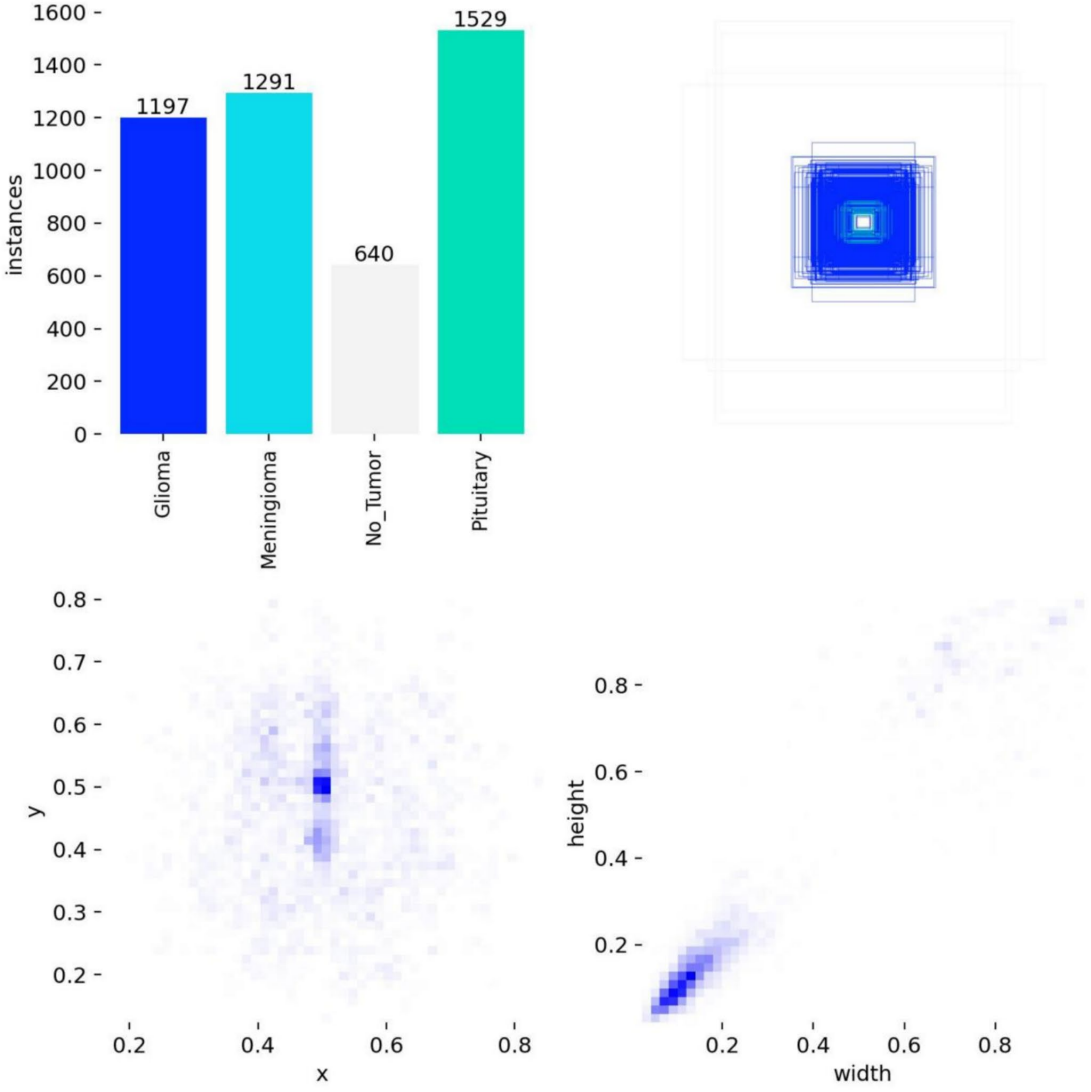
Annotation distribution of the training set.

**Figure 8:**
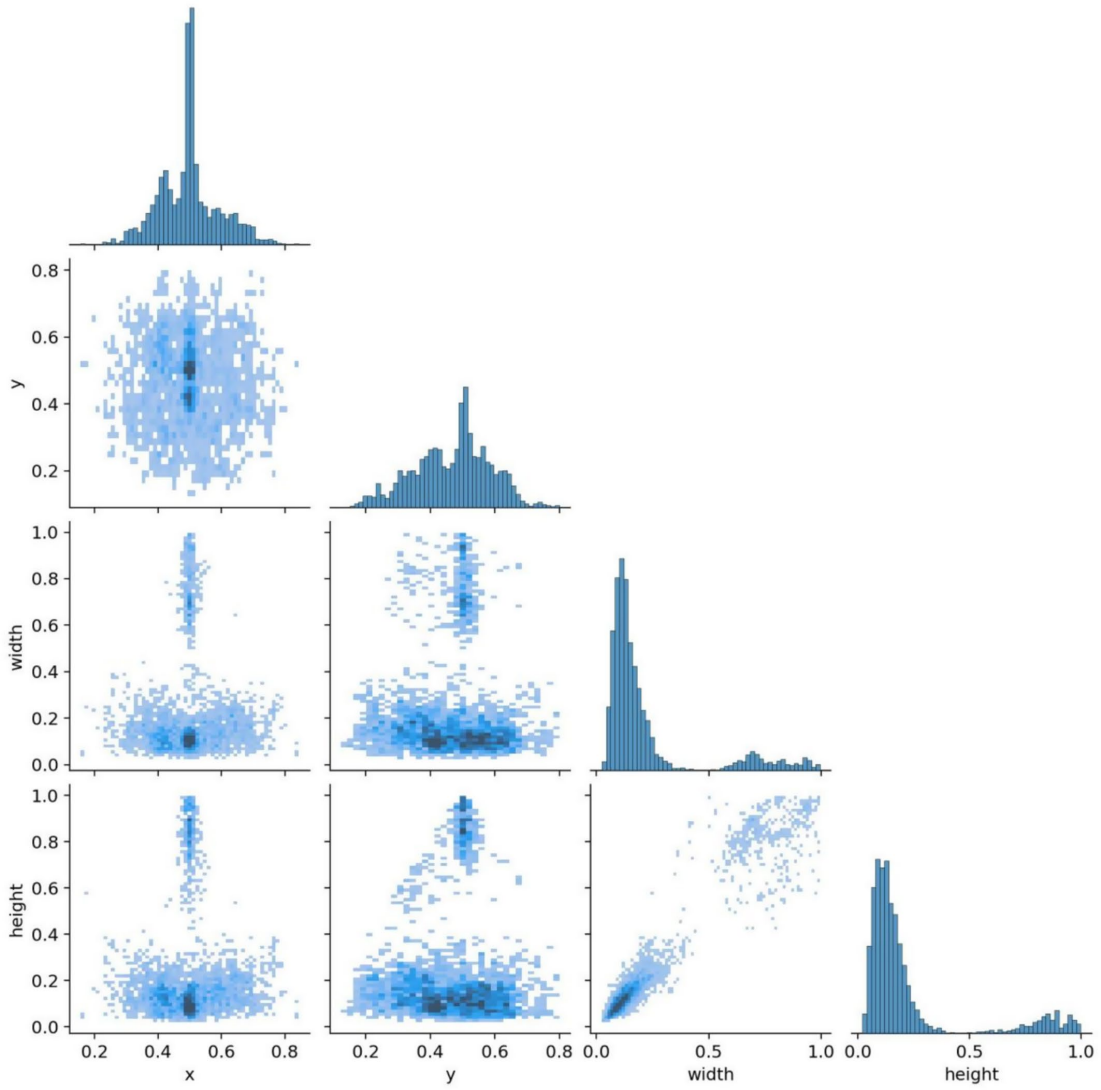
Annotation correlation map of the training set.

All images were pre-annotated with bounding boxes using LabelImg software by two radiologists holding at least intermediate-level professional titles, and the annotations were then reviewed on-site by two associate-senior-or-above radiologists to ensure annotation accuracy. A five-fold cross-validation strategy was employed for model training. From Dataset 1, 5,210 images were randomly sampled and divided into five equal subsets of 1,042 images each; four subsets served as the training set and the remaining one as the validation set, cycled five times to yield five distinct data distribution combinations. The remaining 521 images were reserved as an internal independent test set for evaluating the best-performing model. The training set was used for parameter learning; the validation set independently monitored the training process and triggered early stopping; and the test set was strictly isolated, used exclusively for final performance evaluation. This strategy enables real-time detection of overfitting trends during training, thereby ensuring optimal model performance. The ablation study was evaluated on the validation folds to enable statistical analysis (mean ± standard deviation) across the five cross-validation splits, while the comparative experiments were evaluated on the held-out internal independent test set to ensure a fair and consistent benchmark across all compared models. The annotation distribution characteristics and inter-class correlations of the training dataset are visualized in Figures 7, 8.

### 4.2 Experimental environment

Experiments were conducted on a Windows 11 operating system with an Intel^®^ Core™ Ultra 9275HX CPU, an NVIDIA GeForce RTX 5070 Laptop GPU, 32 GB of RAM, PyTorch 2.8.0, CUDA 12.9, and Python 3.9.23. Model hyperparameters are listed in Table 1. Standard data augmentation techniques were applied during training, including random horizontal flipping, mosaic augmentation, and HSV color jittering. All experiments used a fixed random seed of 0 to ensure reproducibility.

**Table 1:** Model hyperparameter settings.

Image size	Batch size	Epochs	Optimizer	Patience	IoU
640 × 640	8	100	AdamW	10	0.7
Mosaic	hsv_h	hsv_s	hsv_v	Close mosaic	Seed
0.5	0.015	0.7	0.4	10	0

The average training time per fold was approximately 4.8 h for CMRA-DETR and approximately 5.2 h for the baseline RT-DETR-R18 under the above hardware configuration. GPU memory utilization during training was approximately 6.5 GB. The source code and trained model weights will be made publicly available upon acceptance of this manuscript to facilitate independent verification and further research.

### 4.3 Evaluation metrics

A comprehensive set of metrics was employed to evaluate model performance. Detection accuracy was measured by Precision (P), Recall (R), and mean Average Precision (mAP), calculated as shown in Equations (7–10). Model complexity was assessed by parameter count (Params) and floating-point operations (GFLOPs), which reflect memory storage requirements and computational overhead, respectively; lower values indicate a more lightweight model. Inference efficiency was further quantified by frames per second (FPS), measured as the average number of images processed per second during inference, which directly reflects the model’s real-time deployment capability.


$$\begin{array}{c} P=\frac{\mathrm{TP}}{\mathrm{TP}+\mathrm{FP}} \end{array}$$ (7)

$$\begin{array}{c} R=\frac{\mathrm{TP}}{\mathrm{TP}+\mathrm{FN}} \end{array}$$ (8)

$$\begin{array}{c} \mathrm{AP}=\int_{0}^{1}P(R)\mathrm{dR} \end{array}$$ (9)

$$\begin{array}{c} \mathrm{mAP}=\frac{1}{N}\sum_{i=1}^{N}{\mathrm{AP}}_{i} \end{array}$$ (10)

Where TP (True Positive) denotes samples correctly predicted as positive; FP (False Positive) denotes samples incorrectly predicted as positive; FN (False Negative) denotes positive samples incorrectly predicted as negative; and AP (Average Precision) measures the quality of the learned model for a given class. mAP@50 denotes the mean Average Precision across all classes at an intersection-over-union (IoU) threshold of 0.50; mAP@50–95 denotes the mean of the mAP values computed at IoU thresholds ranging from 0.50 to 0.95 in steps of 0.05.

### 4.4 Ablation study

**Figure 9:**
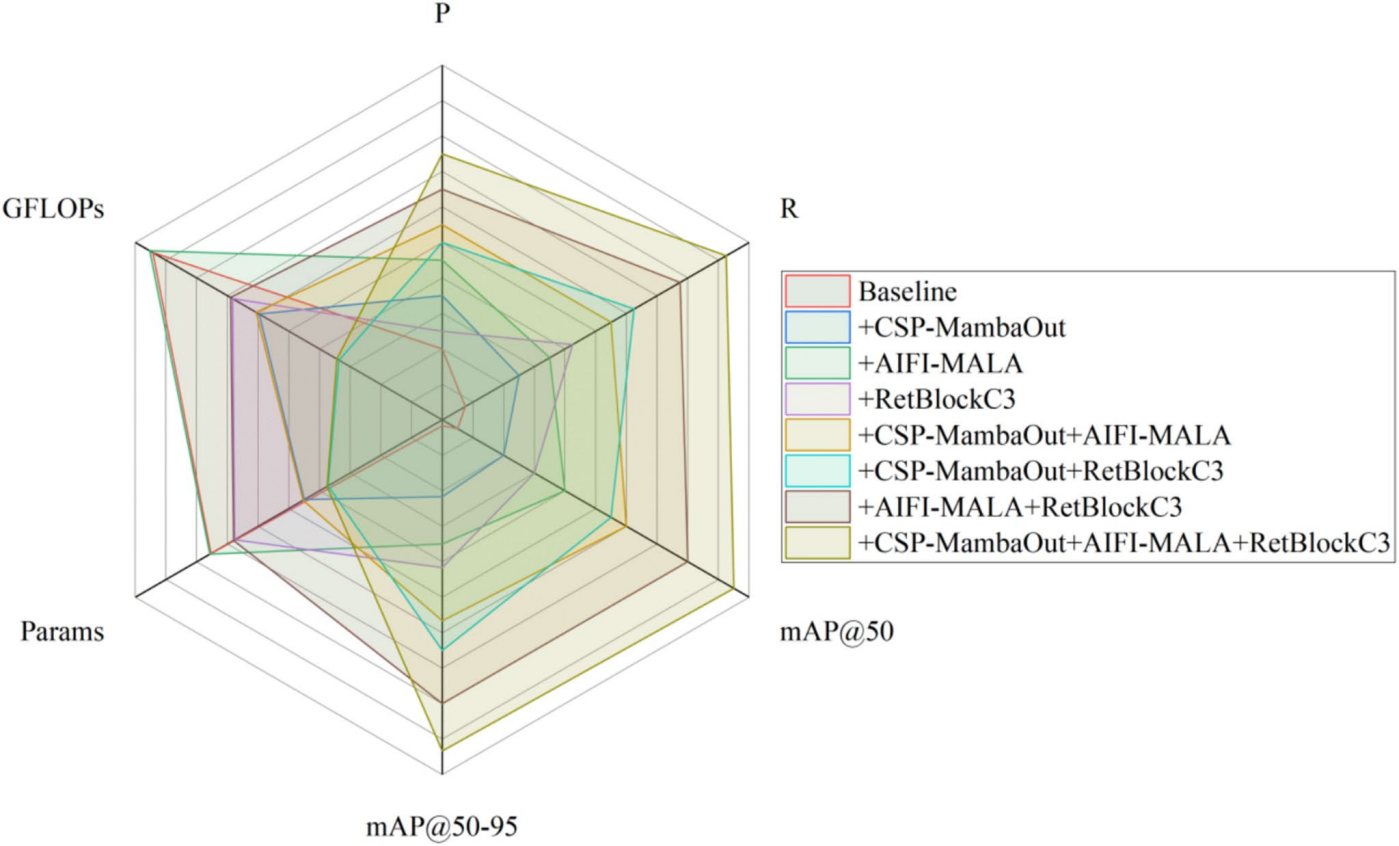
Ablation study comparison.

To verify the independent contribution of each proposed improvement module to model performance, an ablation study was conducted on the validation set of Dataset 1, using the lightweight RT-DETR with a ResNet18 backbone as the baseline. The same hardware environment and hyperparameter settings as the main experiments were employed, combined with a five-fold cross-validation strategy to ensure the reliability and comparability of results. Evaluation metrics included Precision (P), Recall (R), mAP@50, mAP@50–95, parameter count (Params), and GFLOPs. Three primary improvements were introduced incrementally: (i) replacing the original ResNet18 backbone with CSP-MambaOut; (ii) incorporating the AIFI-MALA magnitude-aware feature interaction module into the encoder’s intra-scale interaction module; and (iii) replacing the encoder’s cross-scale aggregation module RepC3 with the RetBlockC3 spatial retention feature aggregation module. Ablation results are shown in Table 2 and Figure 9.

**Table 2:** Ablation experiment results.

CSP-MambaOut	AIFI-MALA	RetBlockC3	P/%	R/%	mAP@50/%	mAP@50–95/%	Params/10^6^	GFLOPs
			93.8 ± 0.5	91.6 ± 0.7	95.4 ± 0.4	76.3 ± 0.6	20.08	58.3
√			94.1 ± 0.4	92.3 ± 0.6	95.8 ± 0.3	77.5 ± 0.5	13.99	47.9
	√		94.3 ± 0.5	92.7 ± 0.5	96.2 ± 0.4	78.3 ± 0.6	20.15	58.6
		√	93.9 ± 0.4	93.0 ± 0.6	96.0 ± 0.3	78.7 ± 0.5	18.53	50.5
√	√		94.5 ± 0.4	93.5 ± 0.5	96.6 ± 0.3	79.6 ± 0.4	14.07	48.2
√		√	94.4 ± 0.3	93.8 ± 0.5	96.5 ± 0.4	80.1 ± 0.5	12.44	40.1
	√	√	94.7 ± 0.4	94.4 ± 0.4	97.0 ± 0.3	81.0 ± 0.4	18.60	50.7
√	√	√	95.0 ± 0.4	95.0 ± 0.5	97.3 ± 0.3	81.8 ± 0.5	12.51	40.3

The ablation results demonstrate that each improvement module independently contributes performance gains, and their combination yields a pronounced synergistic effect. The baseline RT-DETR-R18 achieves *p* = 93.8 ± 0.5%, *R* = 91.6 ± 0.7%, mAP@50 = 95.4 ± 0.4%, and mAP@50–95 = 76.3 ± 0.6% on the validation set, with 20.08 × 10^6^ parameters and 58.3 GFLOPs, providing the reference for subsequent improvements.

When CSP-MambaOut is introduced alone, the parameter count decreases markedly from 20.08 × 10^6^ to 13.99 × 10^6^ (−30.3%) and GFLOPs drop from 58.3 to 47.9 (−17.8%). This demonstrates that the synergistic design of the CSP branch and MambaOut gated feature selection substantially reduces model size while enhancing local perceptual sensitivity to low-contrast lesion textures, validating the effectiveness of this lightweight backbone in medical imaging scenarios. When AIFI-MALA is introduced alone, the parameter count (~20.15 × 10^6^) and GFLOPs (~58.6) remain close to baseline values; however, the magnitude-aware linear attention mechanism, by explicitly modeling feature magnitude information and correcting the distributional smoothing deficiency of standard linear attention, effectively enhances the model’s discriminative sensitivity to lesion signal characteristics and thereby improves detection accuracy. When RetBlockC3 is introduced alone, the parameter count decreases to 18.53 × 10^6^ and GFLOPs to 50.5; the Manhattan distance decay-based spatial retention mechanism introduces an explicit spatial positional prior into cross-scale feature aggregation, strengthening the model’s modeling of spatial continuity for morphologically irregular tumors.

In dual-module combination experiments, the synergistic use of CSP-MambaOut and AIFI-MALA leverages the complementary strengths of lightweight backbone feature extraction and magnitude-aware attention feature enhancement, further improving detection accuracy while keeping parameters and GFLOPs below the baseline. The joint introduction of CSP-MambaOut and RetBlockC3 achieves dual compression of both parameters and computation while accounting for both local texture representation and spatial structure modeling. The combination of AIFI-MALA and RetBlockC3 achieves further improvements in detection accuracy by enhancing the encoder’s feature magnitude discrimination and spatial positional awareness simultaneously. The progressive results across single-, dual-, and three-module combinations collectively reveal a clear performance improvement trend, indicating good synergistic compatibility among the three modules.

With all three improvements integrated, CMRA-DETR achieves the best overall performance: *p* = 95.0 ± 0.4%, *R* = 95.0 ± 0.5%, mAP@50 = 97.3 ± 0.3%, and mAP@50–95 = 81.8 ± 0.5%, representing improvements of 1.1, 3.4, 1.8, and 5.5 percentage points over the baseline, respectively. The particularly pronounced gain in mAP@50–95 indicates a substantial enhancement of detection capability under strict localization accuracy requirements. Simultaneously, the parameter count decreases from 20.08 × 10^6^ to 12.51 × 10^6^ (−37.7%) and GFLOPs from 58.3 to 40.3 (−30.9%), demonstrating that CMRA-DETR achieves notable accuracy improvements alongside substantial model compression, establishing strong deployment potential on resource-constrained devices. Paired t-tests comparing CMRA-DETR’s per-fold mAP@50 against each variant confirm statistically significant improvements (*p* < 0.05) over the baseline (*p* = 0.0021) and all single-module variants (CSP-MambaOut: *p* = 0.0089; AIFI-MALA: *p* = 0.0134; RetBlockC3: *p* = 0.0067). It should be noted that the ablation study was evaluated on validation folds to enable this statistical analysis (mean ± standard deviation), while the comparative experiments in Section 4.5 were evaluated on the held-out independent test set (521 images) to ensure a consistent benchmark. These results validate the rationality and effectiveness of each improvement module.

### 4.5 Comparative experiments

**Figure 10:**
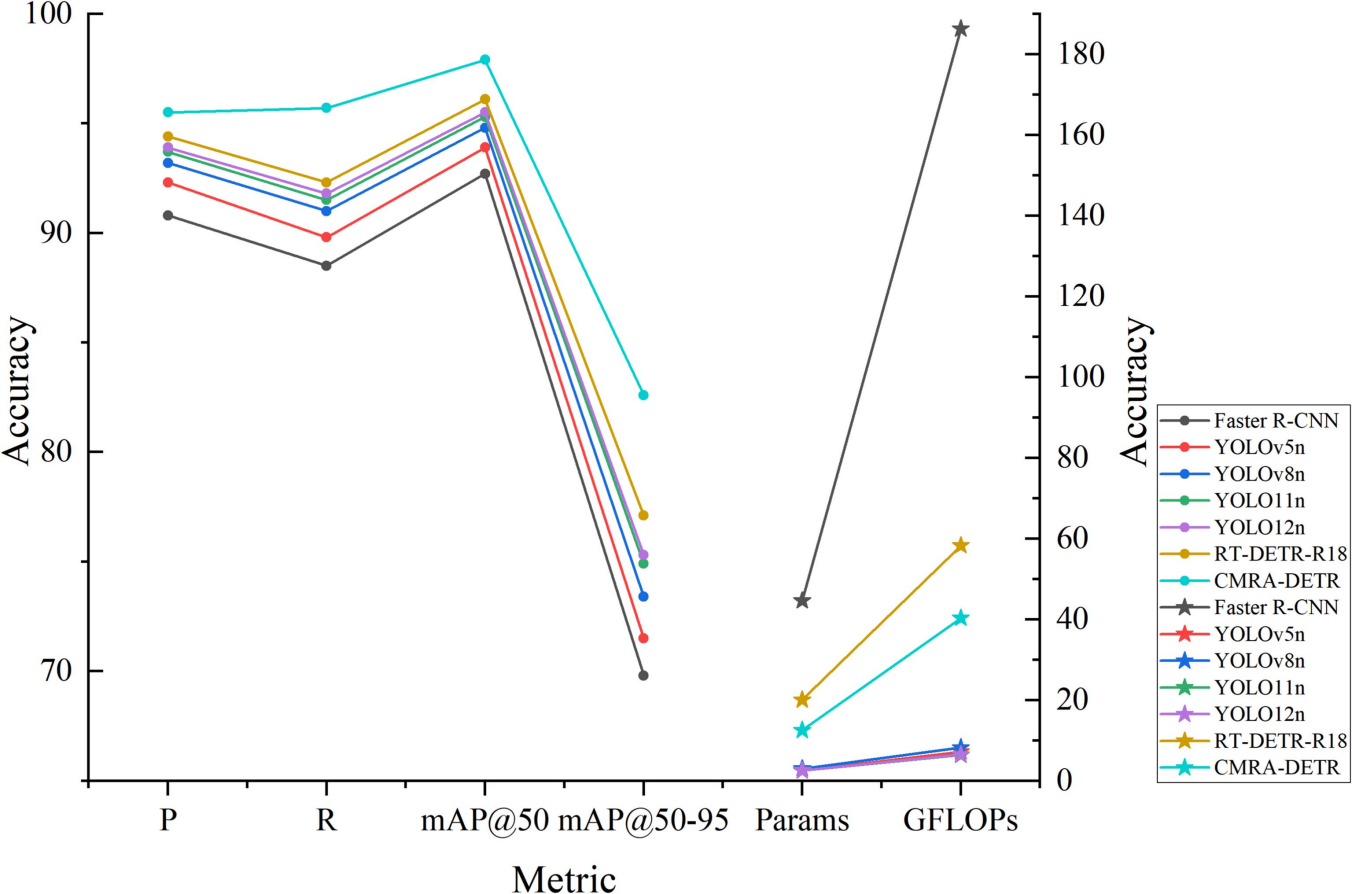
Cross-network experimental comparison.

To further validate the overall performance advantage of CMRA-DETR for brain tumor MRI detection, this section presents a cross-model comparison on the internal independent test set of Dataset 1 against mainstream object detection models, including Faster R-CNN, YOLOv5n, YOLOv8n, YOLO11n, YOLO12n, and the baseline RT-DETR-R18. Since CMRA-DETR is designed for resource-constrained clinical deployment, all baselines are selected within a comparable computational envelope of approximately 2.5–20 M parameters and 6–60 GFLOPs. Full-scale models such as YOLOv8x at 68.2 M parameters or YOLOv9-E at 57.3 M parameters fall outside this envelope, and including them would conflate model capacity with architectural merit rather than reflect practical deployment conditions. Under this constraint, the lightweight or nano variants represent the most competitive baselines available. All comparison models were trained and evaluated under identical dataset splits, training strategies, and hyperparameter configurations to ensure fairness. Inference speed was measured on the same NVIDIA GeForce RTX 5070 Laptop GPU with an input resolution of 640 × 640, using 50 warmup iterations and 300 repeated passes to ensure stable timing. Results are shown in Table 3 and Figure 10.

**Table 3:** Comparative experiment results across different networks.

Model	P/%	R/%	mAP@50/%	mAP@50–95/%	Params/10^6^	GFLOPs	FPS
Faster R-CNN	90.8	88.5	92.7	69.8	44.63	186.3	16
YOLOv5n	92.3	89.8	93.9	71.5	2.51	7.2	148
YOLOv8n	93.2	91.0	94.8	73.4	3.01	8.2	132
YOLO11n	93.7	91.5	95.3	74.9	2.59	6.4	156
YOLO12n	93.9	91.8	95.5	75.3	2.57	6.5	139
RT-DETR-R18	94.4	92.3	96.1	77.1	20.08	58.3	82.7
CMRA-DETR	95.5	95.7	97.9	82.6	12.51	40.3	78.4

As shown in Table 3, CMRA-DETR achieves the highest precision of 95.5% and recall of 95.7% among all compared models. Relative to Faster R-CNN, precision and recall are 4.7 and 7.2 percentage points higher, respectively, while parameter count and computational cost are reduced by 72.0 and 78.4%, and inference speed improves from 16 FPS to 78.4 FPS, confirming the structural advantages of an end-to-end Transformer architecture over the traditional two-stage framework. Compared to the YOLO nano series including YOLOv5n, YOLOv8n, YOLO11n, and YOLO12n, CMRA-DETR surpasses mAP@50 by 2.4–4.0 percentage points and mAP@50–95 by 7.3–11.1 percentage points. The YOLO nano series possesses extremely low parameter counts and computational costs ranging from 2.51 to 3.01 M and 6.4 to 8.2 GFLOPs, and achieves higher throughput ranging from 132 to 156 FPS. However, these models face a clear accuracy ceiling in fine-grained perception tasks such as brain tumor MRI detection, struggling to capture low-contrast lesion boundaries and signal-heterogeneous regions. Compared to the baseline RT-DETR-R18, CMRA-DETR reduces parameter count by 37.7% and GFLOPs by 30.9% while improving mAP@50 by 1.8 percentage points and mAP@50–95 by 5.5 percentage points, with only a minor reduction in inference speed from 82.7 FPS to 78.4 FPS. This marginal throughput decrease is attributable to the additional computation introduced by the proposed modules, yet CMRA-DETR remains well above the real-time threshold of 30 FPS, confirming its suitability for clinical deployment. Overall, with 12.51 M parameters, 40.3 GFLOPs, and 78.4 FPS, CMRA-DETR achieves a favorable balance among detection accuracy, model lightweightness, and inference efficiency, meeting the practical deployment requirements of AI-assisted brain tumor MRI detection under resource-constrained conditions.

**Figure 11:**
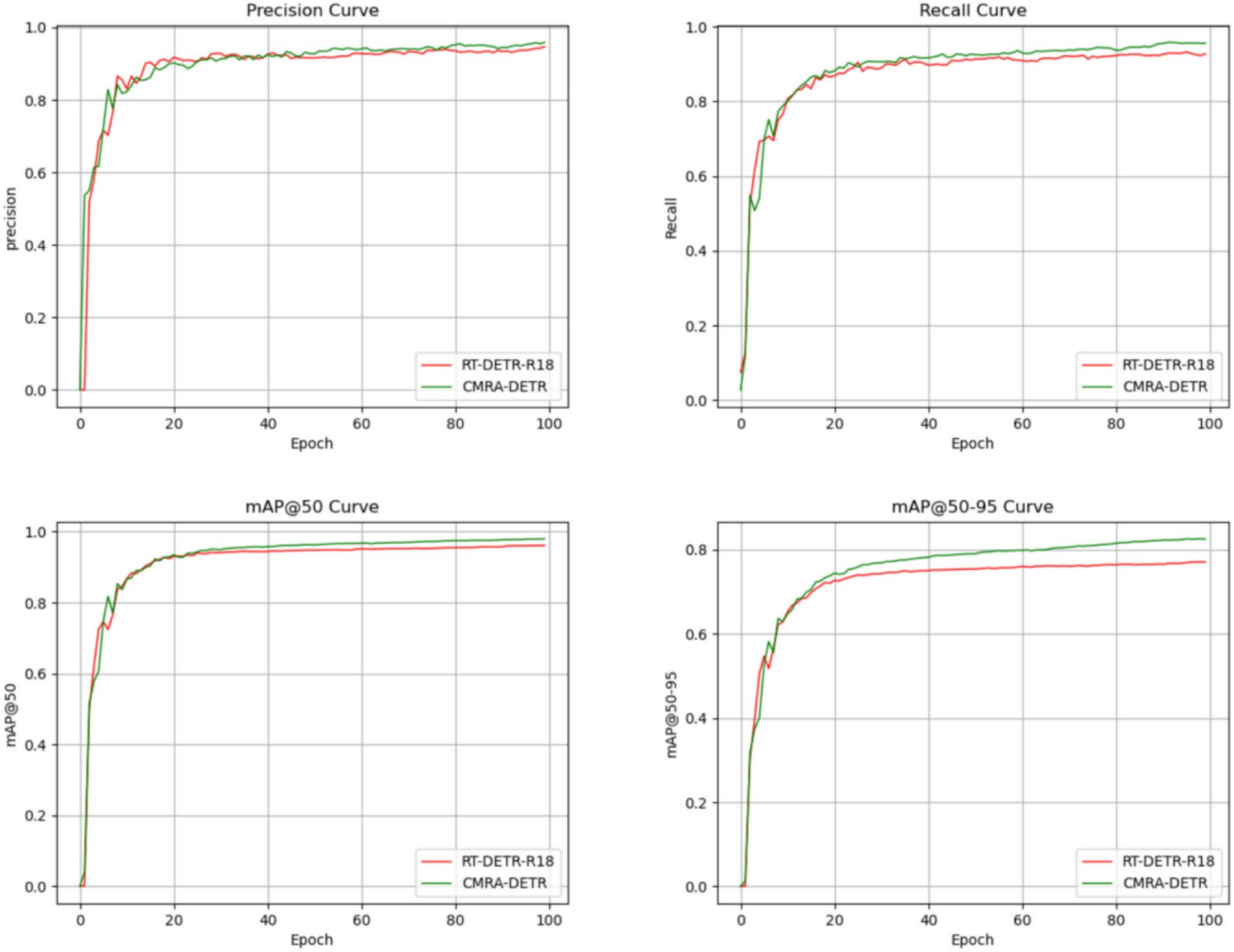
Training curves for precision, recall, mAP@50, and mAP@50–95.

The training curves of precision, recall, mAP@50, and mAP@50–95 are shown in Figure 11. The observed trends indicate that CMRA-DETR achieves improvements across all metrics relative to the baseline model, despite substantially reduced parameter count and computational overhead.

In summary, under the specified experimental conditions, CMRA-DETR achieves competitive or superior performance compared to all evaluated baseline models across all detection metrics, demonstrating a favorable balance among model lightweightness, detection accuracy, and real-time inference capability.

### 4.6 Generalization evaluation

To evaluate the generalization capability of CMRA-DETR for brain tumor MRI detection, Dataset 2, derived from the publicly available BRISC dataset, was used as an external independent test set. This dataset is independently sourced from Dataset 1 and differs substantially in terms of data collection pipeline, subject population, and image characteristics, enabling effective simulation of distribution shift caused by heterogeneous data sources in real-world scenarios, and allowing an objective assessment of model performance on unseen data. CMRA-DETR was evaluated directly using the optimal weights trained on Dataset 1, without any fine-tuning, under evaluation metrics consistent with the preceding experiments. Category-specific metrics and overall model performance are presented in Table 4.

**Table 4:** External test set generalization capability evaluation results.

Category	P/%	R/%	mAP@50/%	mAP@50–95/%
Glioma	93.1	92.4	95.6	77.3
Meningioma	94.2	93.8	96.5	79.8
No_Tumor	95.1	94.9	97.3	81.2
Pituitary	94.8	94.6	97.1	80.9
All	94.3	93.9	96.6	79.8

As shown in Table 4, CMRA-DETR demonstrates strong overall performance on the external independent test set, achieving *p* = 94.3%, R = 93.9%, mAP@50 = 96.6%, and mAP@50–95 = 79.8%. Compared to the internal test set results of mAP@50 = 97.9% and mAP@50–95 = 82.6%, each metric declines by only approximately 1–3 percentage points—a reasonable margin that clearly demonstrates the model’s ability to maintain stable detection performance when facing data from different sources and imaging conditions, effectively validating its cross-dataset generalization capability.

At the category level, the No_Tumor class achieves the highest performance across all metrics, with *p* = 95.1%, R = 94.9%, mAP@50 = 97.3%, and mAP@50–95 = 81.2%, which is expected given the absence of lesion-related visual complexity. The low false-positive rate indicates favorable clinical safety. Among positive categories, Pituitary achieves the best results with mAP@50 = 97.1% and mAP@50–95 = 80.9%, consistent with the typically well-defined boundaries and regular morphology of pituitary adenomas, as well as their characteristic sellar region localization. Meningioma ranks second with mAP@50 = 96.5% and mAP@50–95 = 79.8%, benefiting from relatively distinctive dural attachment patterns and generally stable morphological features. Glioma exhibits the lowest metrics across all indicators, with *p* = 93.1%, *R* = 92.4%, mAP@50 = 95.6%, and mAP@50–95 = 77.3%, though these remain within an acceptable range. This performance gap is attributable to the inherent clinical difficulty of glioma detection. Gliomas, particularly low-grade variants, often present diffuse and irregular boundaries on MRI, leading to greater morphological overlap with surrounding normal tissue. Such ambiguity elevates both false positives, where benign regions are misidentified as glioma, and false negatives, where subtle gliomas are missed by the detector. These findings suggest that enhancing model adaptability for glioma detection across heterogeneous datasets remains a key direction for future improvement.

**Figure 12:**
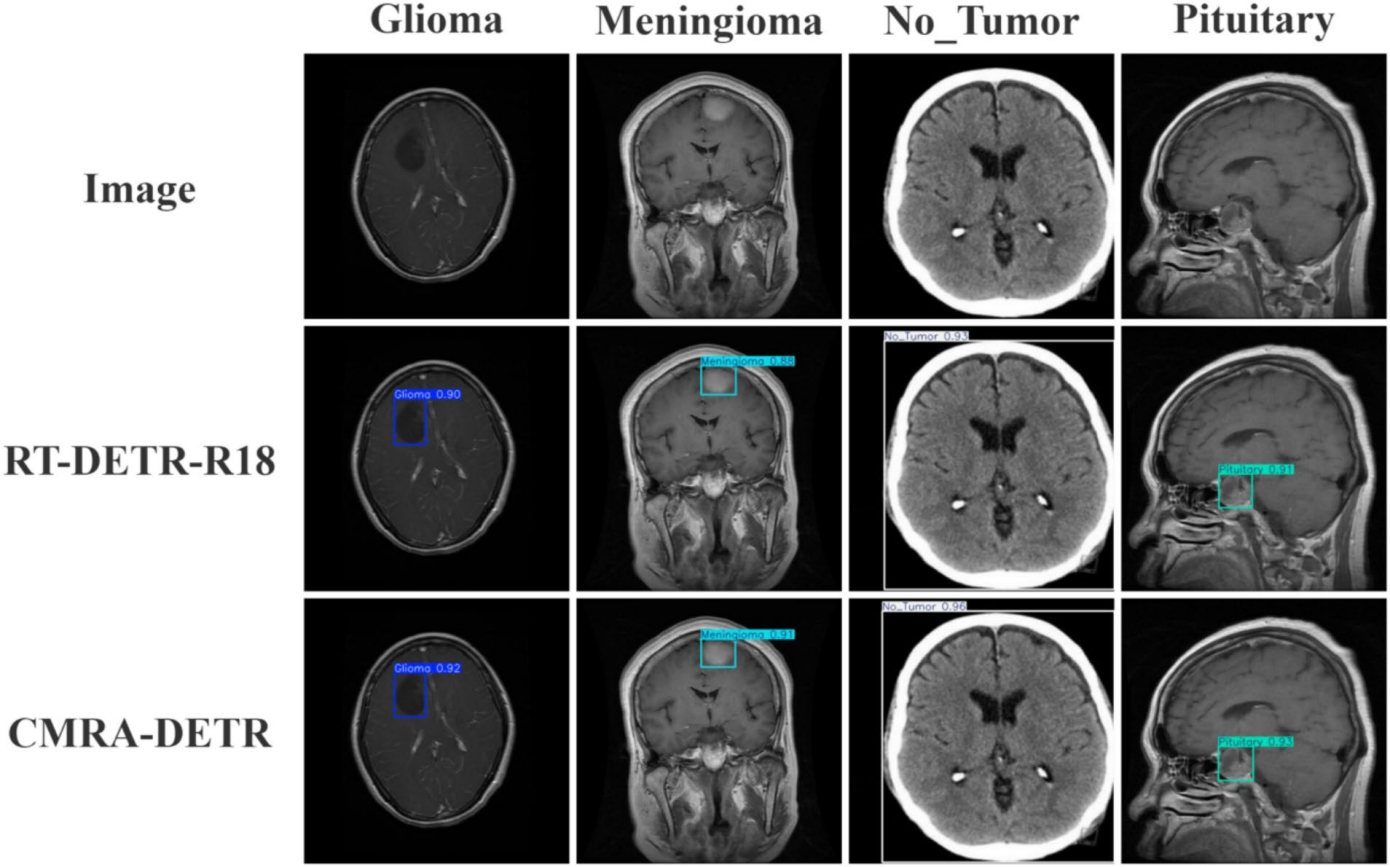
Comparison of detection results before and after model improvement on the external test set.

Figure 12 provides visual detection results of CMRA-DETR on the external independent test set, demonstrating that the model accurately localizes lesions and generates precise bounding boxes with consistent detection quality across pituitary adenoma, meningioma, and glioma cases, further corroborating its cross-dataset generalization capability from a qualitative perspective.

**Figure 13:**
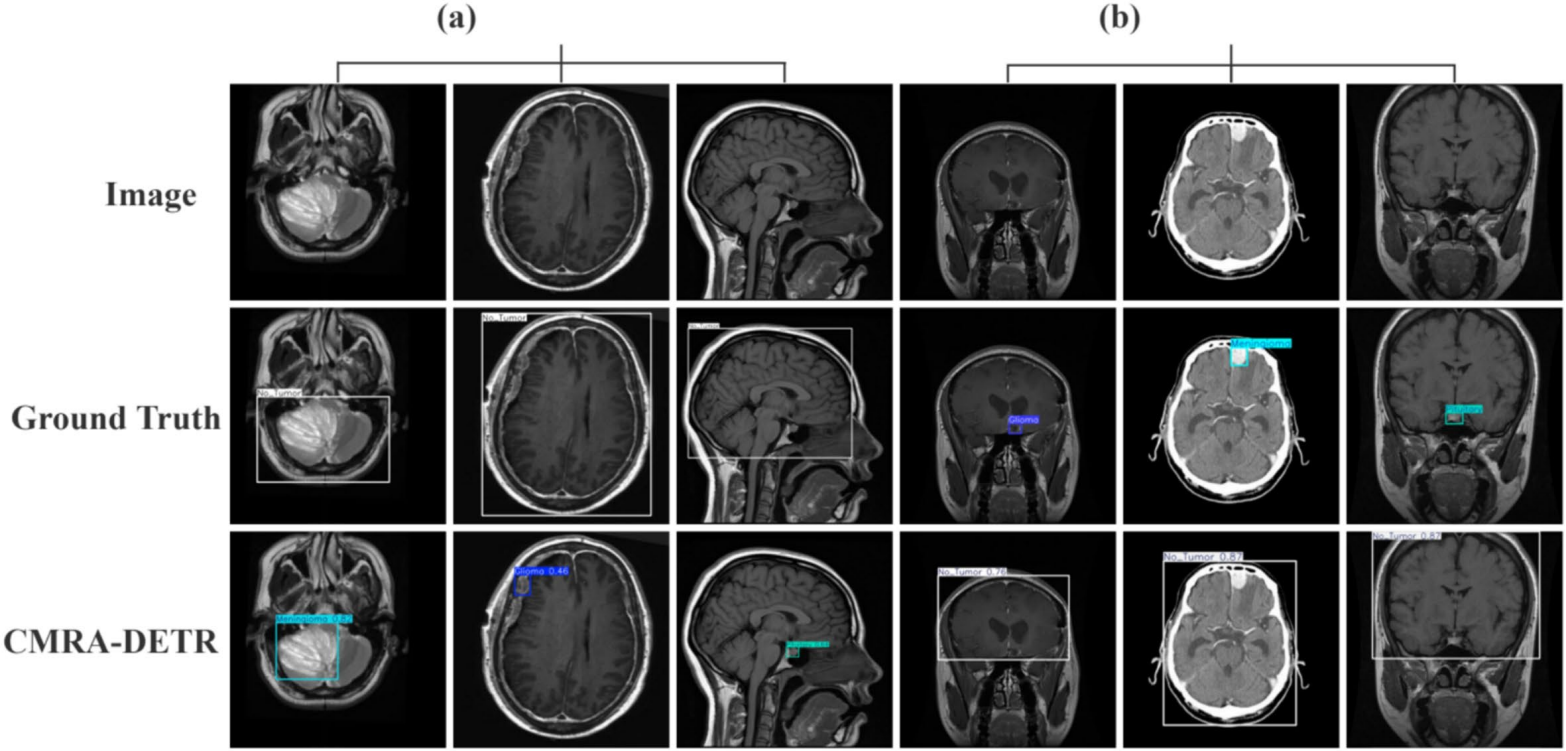
Representative failure cases of CMRA-DETR on the external test set: **(a)** false positive; **(b)** false negative.

To complement the successful detection results shown in Figures 12, 13, presents representative failure cases of CMRA-DETR on the external independent test set, covering two error categories with one example per tumor type. (a) False positive cases: three No-Tumor scans are incorrectly assigned positive labels. A hyperintense posterior fossa structure is misclassified as Meningioma, a normal cortical region triggers a spurious Glioma detection at moderate confidence (0.46), and a sellar-region structure is erroneously predicted as Pituitary. These errors reflect the model’s susceptibility to normal anatomical structures that mimic tumor signal characteristics. (b) False negative cases: one Glioma, one Meningioma, and one Pituitary lesion are each missed and predicted as No-Tumor with high confidence (0.76–0.87), attributed to small lesion size, diffuse boundaries, or limited contrast against surrounding tissue. Together, these failure patterns indicate that signal mimicry and lesion subtlety remain the primary challenges for the model, and they underscore the clinical necessity of radiologist verification in a human-AI collaborative workflow. Addressing these limitations through multi-sequence MRI fusion, boundary-aware loss functions, or uncertainty-guided detection is identified as a priority for future work.

Taken together, the overall performance of CMRA-DETR on the external independent test set closely matches that on the internal test set, indicating that the learned feature representations are highly transferable and that the model has not overfit to the specific distribution of the training data. This demonstrates its potential for deployment across multi-source clinical settings.

### 4.7 Gradient-weighted class activation map visualization

**Figure 14:**
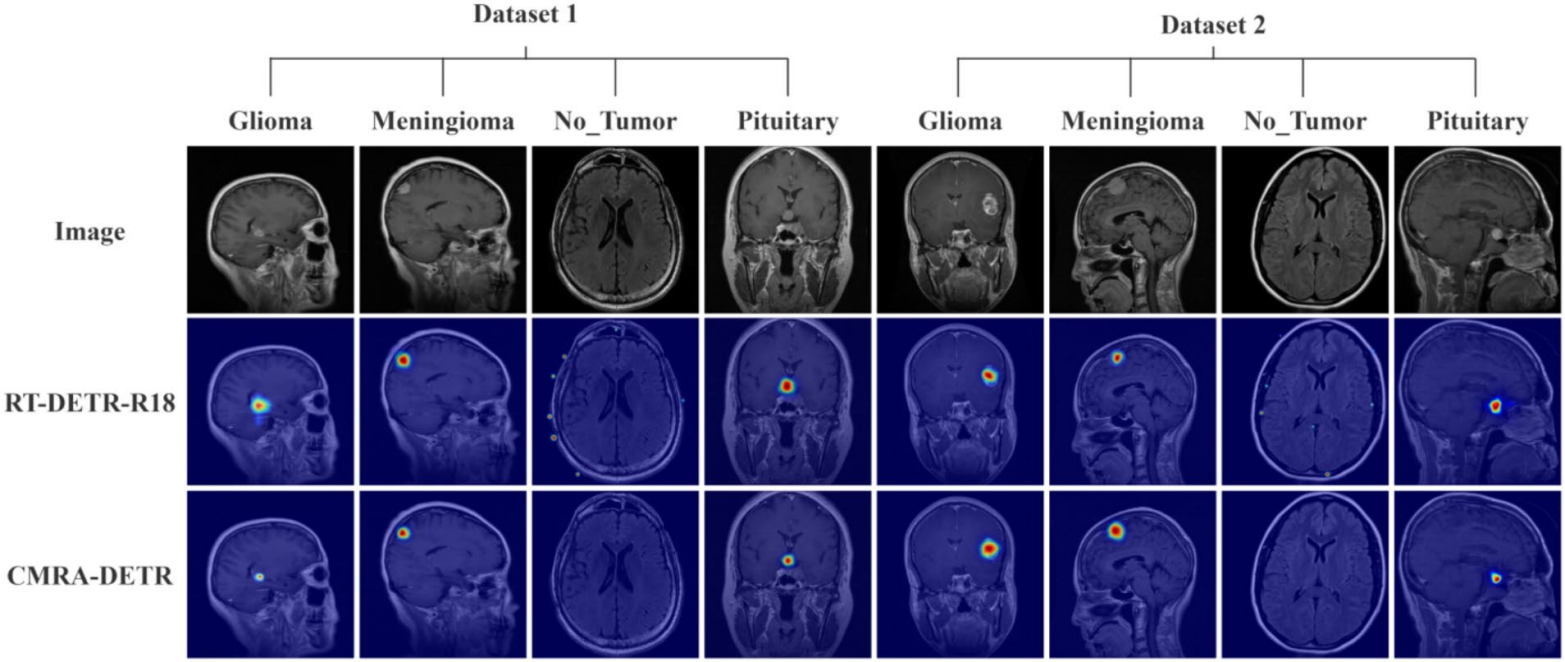
Attention heatmaps for no-tumor versus different tumor types.

To gain deeper insight into the decision-making mechanisms of CMRA-DETR and the lesion attention regions of the model, Gradient-weighted Class Activation Mapping (Grad-CAM) was employed to generate attention heatmaps by computing gradient weights to visualize the model’s focus on different regions of the input image during prediction. In the heatmap visualization analysis, the FPN and PAN multi-scale feature fusion output layers of the detection head were selected as target layers for both the original and improved models: for the baseline model, these correspond to the RepC3 modules at layers 19, 22, and 25; for the improved model, the RetBlockC3 modules at layers 19, 23, and 26. The top 2% of detection results ranked by confidence in descending order were selected as backpropagation targets; gradients were computed by jointly using classification logits and bounding box regression values to generate class activation maps, verifying that model decisions are grounded in tumor-specific lesion regions rather than background noise or irrelevant structures. In the visualizations, red regions represent highly activated areas (i.e., regions of primary model attention), while blue regions indicate low activation. Heatmaps were generated on MRI images from both the internal test set (Dataset 1) and the external independent test set (Dataset 2) to compare the consistency of model attention patterns on in-distribution and out-of-distribution data. The results are presented in Figure 14.

In the heatmap examples shown in Figure 14 (top row: original image; middle row: baseline RT-DETR-R18 heatmap results; bottom row: CMRA-DETR improved model heatmap results), it can be observed that the activation regions of CMRA-DETR are more precisely concentrated on the tumor core and margin regions, with a more compact attention distribution that effectively avoids diffusion of activation energy toward non-lesion background regions. In pituitary adenoma images, the activation center of CMRA-DETR precisely covers the tumor body, with significantly reduced misactivation of adjacent normal pituitary tissue. In meningioma images, the heatmap clearly delineates the tumor boundary contour with effective responses to local features of the intracranial attachment region. In glioma images, the heatmap responses to heterogeneous tumor textures and invasive boundaries are more focused than those of the baseline, indicating stronger discriminative capability for low-contrast, boundary-indistinct lesion features. In no-tumor samples, model activation regions are generally at low levels with no prominent false-positive hotspots, demonstrating that the model effectively suppresses interference from normal brain tissue and provides reliable negative sample classification.

These improvements in activation patterns can be attributed to the synergistic action of the three proposed modules: the CSP-MambaOut backbone, through its gated feature selection mechanism, enhances sensitivity to local textures of low-contrast lesions, enabling accurate lesion signal localization against complex backgrounds; the AIFI-MALA module’s magnitude-aware linear attention explicitly models feature magnitude differences, strengthening the encoder’s discriminative capacity between the signal distributions of lesion and normal tissue, thereby directing attention toward lesion regions; and the RetBlockC3 module’s Manhattan distance decay-based spatial retention mechanism introduces explicit spatial positional priors into feature aggregation, effectively enhancing the model’s modeling of spatial continuity for morphologically irregular tumors and aligning activation boundaries more closely with true lesion contours.

Further comparison of heatmaps between the internal test set and the external independent test set reveals that the activation distribution patterns of CMRA-DETR are highly consistent across both datasets, with stable concentration of attention on lesion core regions and no phenomena such as scattered activations or increased false-positive hotspots caused by distributional shift. This provides additional qualitative evidence of the model’s generalization stability across data sources. The heatmap visualization results not only validate the interpretability of the model’s decision-making process but also offer clinicians an intuitive reference for understanding the basis of AI-assisted diagnostic decisions, facilitating the establishment of clinical trust and promoting practical deployment of the model in real medical settings.

## 5 Discussion

This study proposes CMRA-DETR to address the limitations of RT-DETR in brain tumor MRI detection. The contributions of this work are primarily engineering-oriented rather than theoretically novel: CSP-MambaOut adapts the gated convolutional mechanism of Yu et al. within a CSP framework; AIFI-MALA introduces a magnitude-aware correction into existing linear attention; and RetBlockC3 incorporates the retention mechanism of Fan et al. into the cross-scale aggregation pipeline. The novelty lies not in the individual components themselves, but in their targeted integration and domain-specific adaptation to the unique challenges of brain tumor MRI detection—namely low contrast, indistinct boundaries, and irregular morphology—resulting in a cohesive framework that achieves a favorable accuracy–efficiency balance for resource-constrained clinical deployment.

In the ablation study, the improvement in mAP@50–95 substantially exceeds that in mAP@50, confirming genuine enhancement under stringent localization requirements consistent with the three modules’ design intent. Regarding design rationale: Yu and wang (22) systematically validated that gated convolutional mechanisms can achieve dynamic feature selection without requiring state space models, and CSP-MambaOut is grounded in this principle to enhance local texture perception of low-contrast lesions. Katharopoulos et al. (27) demonstrated that linear attention loses magnitude information after kernel function mapping, resulting in distributional smoothing, and the magnitude-aware correction mechanism of AIFI-MALA directly addresses this deficiency. The core retention mechanism of RetBlockC3 derives from the design approach proposed by Fan et al. (23). Compared with ConvNeXt (large-kernel convolutions) and EfficientNet (compound scaling), CSP-MambaOut’s gated selection is specifically advantageous for low-contrast MRI. MALA addresses the magnitude-smoothing gap of linear attention while maintaining linear complexity. Grad-CAM visualizations (section 4.7) confirm that RetBlockC3 produces more spatially focused activations aligned with tumor boundaries. The reductions in parameter count (−37.7%) and GFLOPs (−30.9%) demonstrate more efficient feature utilization through structural optimization.

Comparative analysis against mainstream models further validates the necessity of domain-specific architectural adaptation. Although the YOLO nano series performs well on general detection tasks, it encounters a clear accuracy ceiling in fine-grained perception scenarios such as brain tumor MRI detection. Litjens et al. (28) noted that direct transfer of general architectures to medical imaging entails challenges including domain shift and intra-class variability. Ferreira et al. (29) demonstrated robust detection under degraded image quality using hybrid YOLO architectures, analogous to clinical MRI conditions. Ferreira et al. (30) reinforced the rationale for domain-specific adaptation. Ferreira et al. (31) emphasized the importance of interpretable decision processes in clinical AI, aligning with the Grad-CAM analysis in section 4.7. Carion et al. (32) and Zhao et al. (18) validated the NMS-free paradigm in RT-DETR. Building upon these foundations, CMRA-DETR achieves consistently favorable performance over Faster R-CNN with a substantially lower parameter count.

CMRA-DETR exhibits only a 1–3 percentage point decrease on the external test set, demonstrating favorable cross-dataset stability. Glocker et al. (33) noted that scanner differences often cause performance degradation in deployed models. The cross-center stability achieved here partially validates the robustness contribution of the proposed improvements. The lower recall for glioma is closely related to its biological characteristics—indistinct boundaries and heterogeneous morphology. As noted by Louis et al. (34) noted that glioma heterogeneity renders their MRI signal distribution highly complex. Future work may incorporate multi-sequence fusion to further improve glioma detection robustness.

This study has several limitations. Maier-Hein et al. (35) noted that single-dataset evaluations are prone to optimistic bias; the Kaggle-sourced training data may not fully capture multi-center clinical variability, and the small external validation set (512 images) limits generalization conclusions. Only four tumor categories are considered, and multi-center validation using BraTS is a key future direction. Additionally, image-level annotation precludes patient-level metrics, boundary segmentation is not addressed, and only a single MRI sequence is used. Bakas et al. (36) systematically demonstrated in the BraTS benchmark that multi-sequence joint modeling (T1, T1ce, T2, FLAIR) provides significant advantages in lesion feature representation; multi-sequence fusion therefore represents an important future extension direction.

Regarding class distribution, the training set exhibits moderate imbalance (meningioma and pituitary adenoma are approximately twice as frequent as no tumor and glioma), which may partially contribute to lower glioma detection performance. Nevertheless, glioma mAP@50 remains above 95% on both test sets. Future work may explore class-aware sampling or focal loss variants to mitigate this issue.

From a clinical deployment perspective, this study lacks clinician-in-the-loop validation, and prospective clinical trials are necessary to assess real-world utility. Both false positives (unnecessary procedures) and false negatives (delayed glioma diagnosis) carry clinical risks requiring thorough cost–benefit analysis. Deployment also requires PACS integration, regulatory compliance, and robustness to protocol variations across institutions. The envisioned scenario is a computer-aided detection tool supporting radiologist review, maintaining the clinician as the final decision-maker in a human–AI collaborative workflow.

In summary, CMRA-DETR achieves a comprehensive balance among detection accuracy, model lightweightness, and generalization capability for brain tumor MRI detection. Future work will focus on three directions: large-scale multi-center validation, multimodal sequence fusion, and real-time inference optimization oriented toward clinical deployment.

## 6 Conclusion

This study presents CMRA-DETR, which addresses three practical limitations of RT-DETR in brain tumor MRI detection—insufficient backbone texture perception, neglect of feature magnitude differences, and weak spatial structure modeling—through the synergistic combination of three improvement modules: CSP-MambaOut, AIFI-MALA, and RetBlockC3. On the internal test set, the model achieves excellent detection accuracy (*p* = 95.5%, *R* = 95.7%, mAP@50 = 97.9%, mAP@50–95 = 82.6%) while reducing parameter count and computational cost by 37.7 and 30.9% relative to the baseline, respectively, achieving competitive or superior performance compared to all evaluated baseline models under the specified experimental conditions. On the external independent test set, the model achieves mAP@50 = 96.6% and mAP@50–95 = 79.8%, further validating its cross-dataset generalization capability and demonstrating practical potential for AI-assisted automated brain tumor MRI detection by radiologists on resource-constrained clinical devices.

## Data Availability

The names of the repository/repositories and accession number(s) can be found below: Brain Tumor MRI Dataset (Dataset 1): Kaggle. https://doi.org/10.34740/KAGGLE/DSV/14832123Brain Tumor Classification (MRI) (Dataset 1): Kaggle. https://doi.org/10.34740/KAGGLE/DSV/12745533BRISC: Annotated Dataset for Brain Tumor Segmentation and Classification (Dataset 2): Scientific Data. https://doi.org/10.1038/s41597-026-06753-y.
